# Digital health information and female adolescents with turkish migration background

**DOI:** 10.1093/eurpub/ckac131.519

**Published:** 2022-10-25

**Authors:** Z Islertas, UH Bittlingmayer

**Affiliations:** Educational University Freiburg Germany, Freiburg, Germany

## Abstract

**Background:**

Youth with Turkish migration background (TRMB) are repeatedly classified as a vulnerable group. Digital health information, which is prepared and made available by various providers in a variety of ways, may represent an opportunity to promote the health of these adolescents. Empirical findings on wheter young people have access to digital health information and what role digital health information plays in their everyday lives are hard to find.

**Methods:**

In the project ELMi, a project of the HLCA Consortium, funded by the BMBF, female adolescents with TRMB were interviewed. Individual interviews as well as a focus group discussion were conducted and evaluated according to the Qualitative Content Analysis.

**Results:**

Female adolescents with TRMB have technical access to digital health information. They use New Media to obtain health information. When searching for digital health information, it is important for the adolescents to be able to find factual and comprehensibly formulated information quickly. German-language homepages are preferred by them. Parallel to the digital platform they regard female persons from their own social network as contact persons for health questions. They draw on their health-related knowledge and experience in order to be able to obtain answers to their own questions.

**Conclusions:**

Digital health information and the health-related knowledge and experience of the female social network are considered as a health related resource. German-language electronic information with low-threshold content can represent an opportunity to reach female adolescents with TRMB

**Key messages:**

• Female adolescents with TRMB demonstrate technical and content access to digital health information.

• Digital health information represents an opportunity to reach the target group.

